# A practical solution of the Bethe equation in the energy range applicable to radiotherapy and radionuclide production

**DOI:** 10.1038/s41598-019-54103-3

**Published:** 2019-11-26

**Authors:** D. M. Martinez, M. Rahmani, C. Burbadge, C. Hoehr

**Affiliations:** 1 Department of Chemical & Biological Engineering, University of British Columbia, Vancouver, V6T 1Z4 Canada; 2 Department of Mathematics, University of British Columbia, Vancouver, V6T 1Z4 Canada; 3 Department of Physics, University of Guelph, Guelph, N1G 2W1 Canada; 4 Life Sciences Division, TRIUMF, Vancouver, V6T 2A3 Canada

**Keywords:** Theoretical nuclear physics, Information theory and computation

## Abstract

While the dose deposition of charged hadrons has received much attention over the last decades starting in 1930 with the publication of the Bethe equation, there are still practical obstacles in implementing it in fields like radiotherapy and isotope production on cyclotrons. This is especially true if the target material consists of non-homogeneous materials, either consisting of a mixture of different elements or experiencing phase changes during irradiation. While Monte-Carlo methods have had great success in describing these more difficult target materials, they come at a computational cost, especially if the problem is time-dependent. This can greatly hinder optimal advancement in therapy and isotope targetry. Here, a regular perturbation method is used to solve the Bethe equation in the limit of small relativistic effects. Particular focus is given to incident energy level relevant to radionuclide production and radiotherapy applications, i.e. 10–200 MeV. We present a series solution for the range and dose distribution in terms of elementary functions, as opposed to special functions which will aid in uptake by practitioners.

## Introduction

Charged hadrons, like ions or more commonly protons, are an increasingly important tool in both radiotherapy, to treat cancer, and in radioisotope production for use in nuclear medicine applications. Currently there are 94 hadron-therapy centers in operation worldwide, with 66 more under construction or in the planning stages^1^, and more than 1270 cyclotrons are currently used for the production of radioisotopes^2^. Understanding the interaction of protons with matter is therefore not restricted to the understanding of fundamental mechanisms, but has also wide-ranging implications for health care^3,4^. Protons differ from photons in how they lose energy while traveling through matter. Charged protons lose energy continuously, mainly through ionization of the atoms of the matter they are traversing. The energy loss per unit distance in the non-relativistic regime was described by Bethe^5^. At the end of their range, before the protons come to rest, the interaction cross–section increases as the proton velocity decreases. This results in the Bragg peak whose position is defined, primarily, by the initial energy of the protons. Knowing this position accurately is crucial when treating cancer to avoid excessive dose to neighboring healthy tissue and to take full advantage of the cell-killing properties of the protons. It is also important when designing targets for medical isotope production, as the Bragg peak coincides with the largest heat deposition. This large localized power deposition, if not properly controlled, can increase the temperature and, in a closed system, the pressure in the target to the point of rupture, leading to the release of radioactivity. While the range of protons in matter is tabulated for several materials^6,7^, it is still difficult to handle for mixed materials, for example non-homogeneous tissue in patients or phase changes in targets. In these situations Monte-Carlo packages like SRIM^8^, FLUKA^9,10^, Geant4^11^, MCNP^12^ and others, are often employed with great success but also significant computational cost and time delay, slowing down development or clinical implementation.

The average energy *E* lost by the protons per unit distance *x* while traversing through matter is given by the Bethe equation^5^ as reported by Grimes *et al*.^13^

1 $$-\frac{dE}{dx}=\frac{4\pi n{z}^{2}}{{m}_{e}{c}^{2}{\beta }^{2}}{(\frac{{e}^{2}}{4\pi {\varepsilon }_{o}})}^{2}(\mathrm{ln}(\frac{2{m}_{e}{c}^{2}{\beta }^{2}}{I(1-{\beta }^{2})})-{\beta }^{2})$$

where *n* is the electron density of the target material; *e* is the electron charge; *m*_*e*_ is the electron mass; *I* is the mean excitation potential of the target material; *z* is the multiple of the electron charge of the projectile; $\beta \equiv u/c$ is the ratio of the projectile velocity to the speed of light in vacuum, *c*; and ${\varepsilon }_{0}$ is the vacuum permittivity. The Bethe equation is a result of a first-order Born approximation with the projectile being faster than the bound electrons in the target atoms.

The energy of a proton beam *E* is related to the mass *m*_*p*_ and velocity of a proton via

2 $$E={m}_{p}{c}^{2}(\gamma -1)\,\,\gamma ={(1-{\beta }^{2})}^{-1/2},$$

and, with the use of the chain-rule, we find

3 $$\frac{dE}{dx}={m}_{p}{\gamma }^{3}u\frac{du}{dx}$$

from which we may express the Bethe equation as

4 $${\gamma }^{3}u\frac{{\rm{d}}u}{{\rm{d}}x}=-\,\frac{A}{{u}^{2}}(\mathrm{ln}(\frac{B{u}^{2}}{1-{\beta }^{2}})-{\beta }^{2}).$$

Here, *A* $({m}^{3}/{s}^{4})$ and *B* $({s}^{2}/{m}^{2})$ are defined as

5 $$A=\frac{4\pi n{z}^{2}}{{m}_{e}{m}_{p}}{(\frac{{e}^{2}}{4\pi {\varepsilon }_{o}})}^{2}\,\,B=\frac{2{m}_{e}}{I}$$

with representative values for common materials given in Table 1. The reduction given by Eqs. 2–5 was advanced by Grimes *et al*.^13^, which we use directly in this work.

**Table 1:** Estimated values for *A* and *B* for the passage of protons using material property values given by^7^.

	*A* (×10^28^) (*m*^3^/*s*^4^)	*B* (×10^−13^) (*s*^2^/*m*^2^)
H_2_	2.20	5.92
He	2.20	2.72
Be	21705	1.79
C_*amorphous*_	26472	1.4
C_*graphite*_	22501	1.46
N_2_	15.44	1.39
O_2_	17.65	1.20
Ne	10.98	0.83
Ar	19.81	0.61
Kr	39.54	0.32
Xe	59.69	0.21
Ga	6970	0.3
H_2_O	1470	1.5
CO_2_	24.43	1.34
CH_4_	11.02	2.73

Using the continuous-slowing-down-approximation (CSDA), Eq. 4 has been integrated from the projectiles’ initial velocity to its resting state using a number of different techniques in order to determine its range *R*, defined as the root of $u(R)=0$^8,14–16^. Of note is a recent solution of the Bethe equation advanced by Grimes *et al*.^13^ who show that in the non-relativistic limit ($\varepsilon \equiv {u}_{o}/c\to 0$)

6 $$\frac{RA}{{u}_{o}^{4}}=-\,\frac{1}{2{b}^{2}}{E}_{1}(\,-\,2\,\mathrm{ln}(b))+O({\varepsilon }^{2})\,\,(b\equiv B{u}_{o}^{2}\,{\rm{with}}\,b > 1)$$

where *E*_1_ is the exponential integral; *u*_*o*_ is the initial velocity of the particles; *b* is dimensionless and related to the ratio of the incident energy to the mean excitation potential; and the big-*O* notation is used to describe the error term in the approximation. It indicates that the absolute value of the error is approximately ${\varepsilon }^{2}$. We also note that the grouping on the left-hand side of the equation is dimensionless indicating that the characteristic length ${\ell }_{c}$ for this problem is ${\ell }_{c}\equiv {u}_{o}^{4}/A$, as *R* has units of length. The third dimensionless group that governs this problem is $\varepsilon$, the ratio of the particle velocity to the speed of light. This solution indicates that the (dimensionless) range $R/{\ell }_{c}$ may be uniquely expressed as a function of *b* and $\varepsilon$, and to lowest order is independent of relativistic effects. More importantly, this solution methodology may be extended and used to estimate the dose distribution. However, to do so one requires the use of an inverse exponential integral function, a non-standard function, which Grimes *et al*.^13^ indicate that no closed-form expression exists which hinders the utility of this expression. Overcoming this difficulty motivates this work.

In this work, we present a solution of the Bethe equation using a regular perturbation method. Using a solution methodology different to that performed by Grimes *et al*.^13^, we are able to create a reasonable solution which avoids the use of the exponential integral. We present our solution in the small relativistic limit and then extend the number of terms to cover the energy range up to ~200 MeV for protons. We find that with this novel solution the complexity of using the Bethe equation has been reduced dramatically, without significant loss of precision. Because of this, we anticipate uptake of these expressions by the radioisotope and radiotherapy communities^17^ for optimization purposes, for example target design.

## Model Derivation

Our methodology is inspired by Grimes *et al*.^13^ who demonstrate that the problem can be approximated by a series solution. Here, we present an alternative methodology in creating the series and begin our solution by scaling Eq. 4 with

7 $${\boldsymbol{u}}=\frac{u}{{u}_{o}}\,\,({\boldsymbol{x}},{\boldsymbol{R}})=\frac{1}{{\ell }_{c}}(x,R)$$

and then switch the roles of the dependent and independent variables to avoid divergence of the velocity gradient near the Bragg peak. This yields

8 $$\frac{{\rm{d}}{\boldsymbol{x}}}{{\rm{d}}{\boldsymbol{u}}}=-\,\frac{{{\boldsymbol{u}}}^{3}}{{(1-{(\varepsilon {\boldsymbol{u}})}^{2})}^{3/2}}{(\mathrm{ln}(-\frac{b{{\boldsymbol{u}}}^{2}}{1-{(\varepsilon {\boldsymbol{u}})}^{2}})-{(\varepsilon {\boldsymbol{u}})}^{2})}^{-1}\equiv {\mathfrak{F}}({\boldsymbol{u}}),\,\,{\boldsymbol{x}}(1)=0$$

where ${\mathfrak{F}}({\boldsymbol{u}})$ can be approximated by a Taylor series about $\varepsilon =0$, i.e.

9 $$\begin{array}{rcl}{\mathfrak{F}}({\boldsymbol{u}}) & = & -\frac{{{\boldsymbol{u}}}^{3}}{\mathrm{ln}(b{{\boldsymbol{u}}}^{2})}-{\varepsilon }^{2}\frac{3{{\boldsymbol{u}}}^{5}}{2\,\mathrm{ln}\,(b{{\boldsymbol{u}}}^{2})}\\  &  & -\,{\varepsilon }^{4}\frac{{{\boldsymbol{u}}}^{7}}{\mathrm{ln}\,(b{{\boldsymbol{u}}}^{2})}(\frac{15}{8}-\frac{1}{2\,\mathrm{ln}\,(b{{\boldsymbol{u}}}^{2})})\\  &  & -\,{\varepsilon }^{6}\frac{{{\boldsymbol{u}}}^{9}}{\mathrm{ln}\,(b{{\boldsymbol{u}}}^{2})}(\frac{35}{16}-\frac{13}{12\,\mathrm{ln}\,(b{{\boldsymbol{u}}}^{2})})+O({\varepsilon }^{8})\end{array}$$

If we seek a solution of the form

10 $${\boldsymbol{x}}({\boldsymbol{u}})={{\boldsymbol{x}}}_{o}({\boldsymbol{u}})+{\varepsilon }^{2}{{\boldsymbol{x}}}_{1}({\boldsymbol{u}})+{\varepsilon }^{4}{{\boldsymbol{x}}}_{2}({\boldsymbol{u}})+{\varepsilon }^{6}{{\boldsymbol{x}}}_{3}({\boldsymbol{u}})+O({\varepsilon }^{8})$$

and reduce the complexity of the notation by transforming the dependent variable using

11 $$z=-\,2\,\mathrm{ln}(b{{\boldsymbol{u}}}^{2})$$

we find that

12 $$\begin{array}{ll}O(1): & \frac{{\rm{d}}{{\boldsymbol{x}}}_{0}}{{\rm{d}}z}=-\,\frac{1}{2{b}^{2}}\frac{{{\rm{e}}}^{-z}}{z}\end{array}$$

13 $$\begin{array}{ll}O({\varepsilon }^{2}): & \frac{{\rm{d}}{{\boldsymbol{x}}}_{1}}{{\rm{d}}z}=-\,\frac{3}{4{b}^{3}}\frac{{{\rm{e}}}^{-\frac{3z}{2}}}{z}\end{array}$$

14 $$\begin{array}{ll}O({\varepsilon }^{4}): & \frac{{\rm{d}}{{\boldsymbol{x}}}_{2}}{{\rm{d}}z}=-\,\frac{1}{16{b}^{4}}\frac{{{\rm{e}}}^{-2z}(15\,z+8)}{{z}^{2}}\end{array}$$

15 $$\begin{array}{ll}O({\varepsilon }^{6}): & \frac{{\rm{d}}{{\boldsymbol{x}}}_{3}}{{\rm{d}}z}=-\,\frac{{{\rm{e}}}^{-5/2z}(105\,z+104)}{96\,{b}^{5}{z}^{2}}\end{array}$$

when terms of equal order are equated. Under this transform, we note that at the stopping distance the transformed variable is unbounded, *i*.*e*. $z\to \infty$, as ${\boldsymbol{u}}\to 0$. Indeed, when the beam first enters the target ${z}_{o}=-\,2\,\mathrm{ln}(b)$ as ${\boldsymbol{u}}(0)=1$.

## Applications

### Estimating range

We estimate the range by solving Eq. 12 as an example. Following this we simply report the solution for the higher order terms, i.e. Eqs. 13 and 15. We begin by integrating Eq. 12 by parts to give

16 $${\boldsymbol{R}}=-\,\frac{1}{2{b}^{2}}\,{\int }_{{z}_{o}}^{\infty }\,\frac{{{\rm{e}}}^{-{\rm{z}}}}{z}dz=-\,\frac{1}{2{b}^{2}}({-\frac{{{\rm{e}}}^{-z}}{z}|}_{{z}_{o}}^{\infty }-{\int }_{{z}_{o}}^{\infty }\,\frac{{{\rm{e}}}^{-z}}{{z}^{2}}dz)=-\,\frac{1}{2{b}^{2}}(\frac{{{\rm{e}}}^{-{z}_{o}}}{{z}_{o}}-{\int }_{{z}_{o}}^{\infty }\,\frac{{{\rm{e}}}^{-z}}{{z}^{2}}dz)+O({\varepsilon }^{2})$$

In an unmotivated trick, we do so so again and find that

17 $${\boldsymbol{R}}=-\,\frac{1}{2{b}^{2}}(\frac{{{\rm{e}}}^{-{z}_{o}}}{{z}_{o}}-\frac{{{\rm{e}}}^{-{z}_{o}}}{{z}_{o}^{2}}+2\,{\int }_{{z}_{o}}^{\infty }\,\frac{{{\rm{e}}}^{-z}}{{z}^{3}}dz)+O({\varepsilon }^{2})$$

With this we postulate that the solution of the problem may be expressed as a series in 1/*z*_*o*_ as the magnitude of *z*_*o*_ is large. When this integration procedure is repeated, we find that

18 $${\boldsymbol{R}}=-\,\frac{1}{2{b}^{2}}\frac{{e}^{-{z}_{o}}}{{z}_{o}}(1-\frac{1}{{z}_{o}}+\frac{2}{{z}_{o}^{2}}+\ldots )=-\,\frac{{e}^{-{z}_{o}}}{2{b}^{2}}\,\mathop{\sum }\limits_{n=1}^{N}\,{(-1)}^{n+1}\frac{(n-1)!}{{z}_{o}^{n}}$$

For insight into this series, we estimate the magnitude of the three first terms using helium as the target material and 100 MeV protons as the projectiles. We estimate $b=4.5\times {10}^{3}$ and ${z}_{0}=-\,16.8$, giving *i*.*e*. $-\,1/{z}_{0}=0.06$ and $2/{z}_{0}^{2}=0.007$; hence $1 < -\,1/{z}_{0} < 2/{z}_{0}^{2}$, implying a convergent series. This solution may be expressed in a more convenient form by using Eq. 11. Indeed, we find that

19 $${\boldsymbol{R}}=\frac{2\,{\mathrm{ln}}^{2}\,b+\,\mathrm{ln}\,b-1}{8\,{\mathrm{ln}}^{3}\,b}$$

when truncated after three terms, representing an error of ~1% at 100 MeV. We extend the leading-order solution by repeating the procedure given above and solve Eqs. 13–15 using the same procedure to find that

20 $$\begin{array}{rcl}{\boldsymbol{R}} & = & (\frac{2\,{\mathrm{ln}}^{2}\,b+\,\mathrm{ln}\,b-1}{8\,{\mathrm{ln}}^{3}\,b})+{\varepsilon }^{2}(\frac{9\,{\mathrm{ln}}^{2}\,b-3\,\mathrm{ln}\,b+2}{36\,{\mathrm{ln}}^{3}\,b})\\  &  & +\,{\varepsilon }^{4}(\frac{120\,{\mathrm{ln}}^{2}\,b-2\,\mathrm{ln}\,b-1}{512\,{\mathrm{ln}}^{3}\,b})-31{\varepsilon }^{6}(\frac{25\,{\mathrm{ln}}^{2}\,b+5\,\mathrm{ln}\,b+2}{2400\,{\mathrm{ln}}^{3}\,b})\end{array}$$

**Figure 1:**
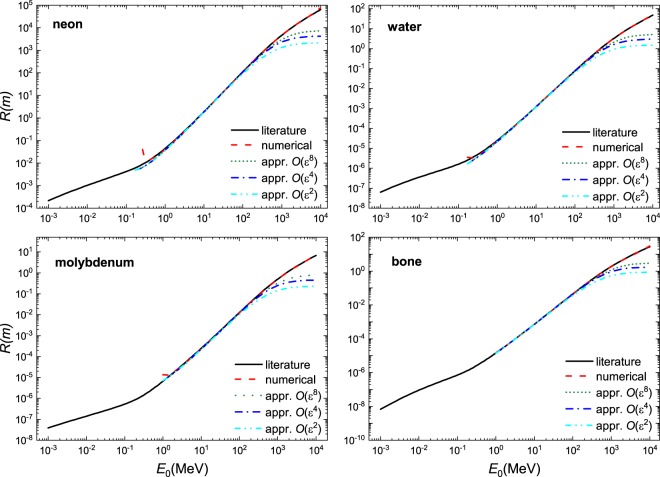
A comparison of solution of the numerical and analytical solutions of Bethe Eq. 20 to literature data^7^ for four different materials: gas (Ne), liquid (H_2_O), solid (Mo), and a complex solid (bone). The results are presented in dimensional form *R* as defined in Eq. 7.

**Figure 2:**
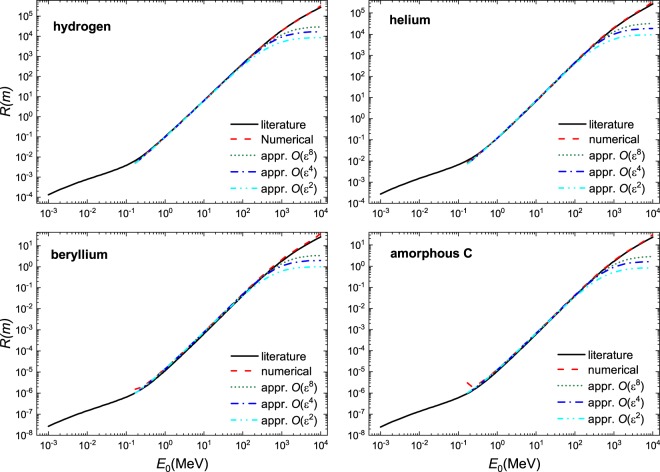
A comparison of solution of the numerical and analytical solutions of Bethe equation Eq. 20 to literature data^7^ for four different materials: Hydrogen (H_2_), Helium (He), Beryllium (Be), and amorphous carbon (C). The results are presented in dimensional form *R* as defined in Eq. 7.

**Figure 3:**
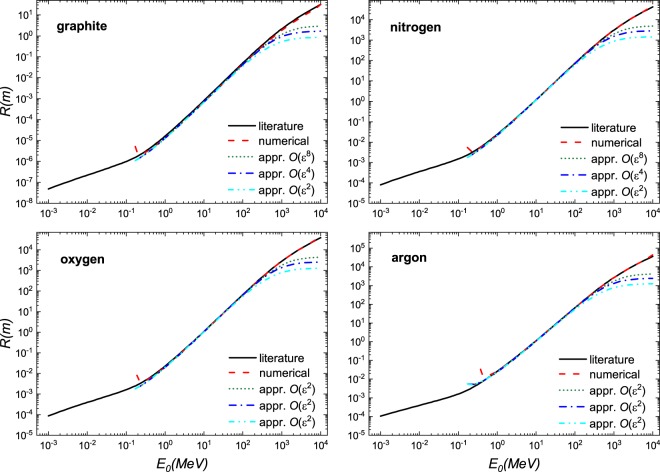
A comparison of solution of the numerical and analytical solutions of Bethe Eq. 20 to literature data^7^ for four different materials: graphite (C), Nitrogen (N_2_), Oxygen (O_2_), and Argon (Ar). The results are presented in dimensional form *R* as defined in Eq. 7.

**Figure 4:**
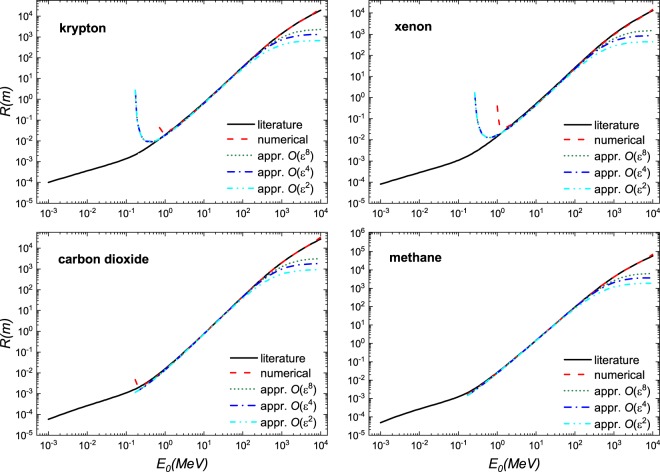
A comparison of solution of the numerical and analytical solutions of Bethe equation Eq. 20 to literature data^7^ for four different materials: Krypton (Kr), Xenon (Xe), Carbon Dioxide (CO_2_), and Methane (CH_4_). The results are presented in dimensional form *R* as defined in Eq. 7.

**Figure 5:**
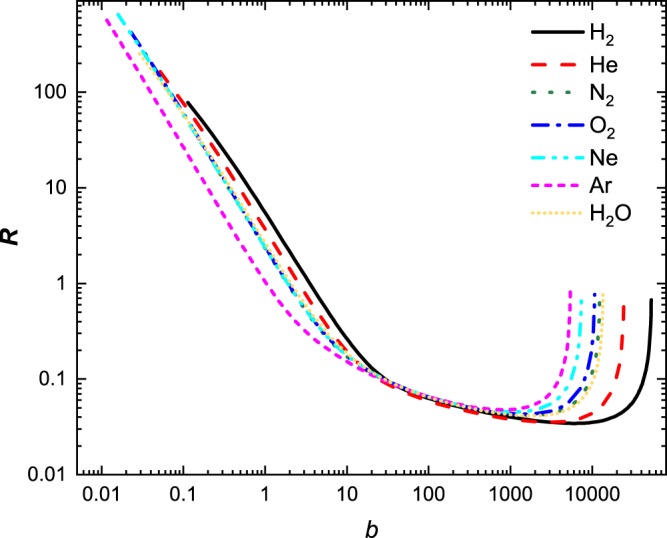
A comparison of solution of the numerical and analytical solutions of Bethe Eq. 20 to literature data^7^. The data is presented in dimensionless form ***R*** as defined in Eq. 7.

We demonstrate the usefulness of this solution in Fig. 1 where excellent agreement is found when compared to literature values^7^ and a numerical solution of the Bethe equation using Romberg integration by applying Richardson extrapolation on a trapezoidal rule for a select number of materials - a gas, liquid, a solid and a complex material; a larger selection of material is shown in Figs. 2, 3 and 4. These calculations were performed using the parameters given in Table 1. At $O({\varepsilon }^{2})$ the solution is fairly reasonable to 50 MeV; at $O({\varepsilon }^{8})$ the expression yields reasonable estimates to ~200 MeV. The immediate benefit of Eq. 20 is that we are able to estimate the stopping distance without use of the exponential integral. Increasing the number of terms does not improve the fit below 1 MeV as the Barkas and Bloch corrections are not included. Interestingly, we find that the empirical expression^18,19^

21 $$R=\alpha {E}_{0}^{p}$$

behaves similarly to our solution. Here, *α* is a material dependent constant and *p* depends on the proton velocity which typical ranges from 1.5 to 2. As can be seen in Fig. 5, in the region ${10}^{1} < b < {10}^{3}$, *R* varies only weakly implying that over small energy intervals, *R* may be taken as constant, yielding $R\propto {E}_{0}^{2}$. This is also evident if we solve the Bethe equation without including the logarithmic term.

### Estimating dose distribution

To estimate the dose distribution ***u***(***x***), we proceed in an analogous manner to that given above. Here, we examine the solution to the leading order equation, i.e. Eq. 12 and then report the solution to the higher order terms. As Eq. 12 is separable, we are in a position to integrate this expression by parts, repeatedly, to generate a series in 1/*z*. We abandon the initial condition (${\boldsymbol{x}}({z}_{o})=0$) and in its place, use the stopping condition defining the range,(${\boldsymbol{x}}({z}_{o}\to \infty )={\boldsymbol{R}}$). By doing so, this dramatically aids in the evaluation of the integral and allows for the development of the series. To highlight this procedure, we rewrite Eq. 12 as

22 $${\int }_{{\boldsymbol{x}}}^{{\boldsymbol{R}}}\,d{\boldsymbol{x}}=-\,\frac{1}{2{b}^{2}}\,{\int }_{z}^{\infty }\,\frac{{{\rm{e}}}^{-z}}{z}dz+O({\varepsilon }^{2})$$

which can be integrated to give

23 $${\boldsymbol{R}}-{\boldsymbol{x}}=-\,\frac{1}{2{b}^{2}}({-\frac{{{\rm{e}}}^{-z}}{z}|}_{z}^{\infty }-{\int }_{z}^{\infty }\,\frac{{{\rm{e}}}^{-z}}{{z}^{2}}dz)=-\,\frac{1}{2{b}^{2}}(\frac{{{\rm{e}}}^{-z}}{z}-{\int }_{z}^{\infty }\,\frac{{{\rm{e}}}^{-z}}{{z}^{2}}dz)+O({\varepsilon }^{2})$$

This is directly analogous to the problem solved previously and allows us to write

24 $${\boldsymbol{R}}-{\boldsymbol{x}}=-\,\frac{{e}^{-z}}{2{b}^{2}}\,\mathop{\sum }\limits_{n=1}^{N}\,{(-1)}^{n+1}\frac{(n-1)!}{{z}^{n}}$$

or

25 $${\boldsymbol{x}}-{\boldsymbol{R}}=-\,\frac{{{\boldsymbol{u}}}^{4}(2{(\mathrm{ln}(b{{\boldsymbol{u}}}^{2}))}^{2}+\,\mathrm{ln}(b{{\boldsymbol{u}}}^{2})+1)}{8{(\mathrm{ln}(b{{\boldsymbol{u}}}^{2}))}^{3}}$$

when truncated after three terms. When this process is repeated and extended to the next term we find

26 $${\boldsymbol{x}}-{\boldsymbol{R}}=-\,\frac{{{\boldsymbol{u}}}^{4}(2{(\mathrm{ln}(b{{\boldsymbol{u}}}^{2}))}^{2}+\,\mathrm{ln}(b{{\boldsymbol{u}}}^{2})+1)}{8{(\mathrm{ln}(b{{\boldsymbol{u}}}^{2}))}^{3}}-{\varepsilon }^{2}\frac{{{\boldsymbol{u}}}^{6}(9{(\mathrm{ln}(b{{\boldsymbol{u}}}^{2}))}^{2}+3\,\mathrm{ln}(b{{\boldsymbol{u}}}^{2})+2)}{36{(\mathrm{ln}(b{{\boldsymbol{u}}}^{2}))}^{3}}$$

Finally, we apply the solution methodology to the higher order terms as

27 $$\begin{array}{ll}O({\varepsilon }^{4}): & -\frac{(120{(\mathrm{ln}(b{{\boldsymbol{u}}}^{2}))}^{2}-2\,\mathrm{ln}(b{{\boldsymbol{u}}}^{2})-1){{\boldsymbol{u}}}^{8}}{512{(\mathrm{ln}(b{{\boldsymbol{u}}}^{2}))}^{3}}\end{array}$$

28 $$\begin{array}{ll}O({\varepsilon }^{6}): & +\frac{31(25{(\mathrm{ln}(b{{\boldsymbol{u}}}^{2}))}^{2}+5\,\mathrm{ln}(b{{\boldsymbol{u}}}^{2})+2){{\boldsymbol{u}}}^{10}}{2400{(\mathrm{ln}(b{{\boldsymbol{u}}}^{2}))}^{3}}\end{array}$$

**Figure 6:**
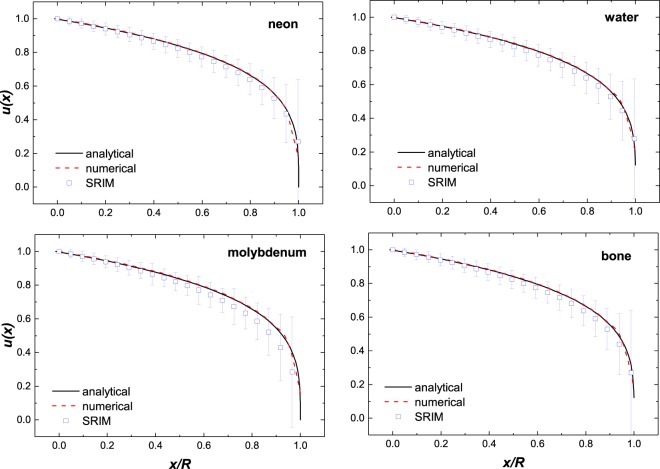
A comparison of solution of the numerical and analytical solutions of Bethe equation Eq. 26 at 100 MeV protons for four different materials: gas (Ne), liquid (H_2_O), solid (Mo), and a complex solid (bone). The data points are results from SRIM^8^.

A representative comparison is given in Fig. 6 where the numerical and approximate solution are compared to SRIM^8^ of the dose distribution of 100 MeV protons impinging in four different materials. The data points are average energy results of protons intersecting the individual depth and the error bars are the corresponding standard deviations of the individual energy spreads. Again, very good agreement is observed between the analytical and our approximate solution and with SRIM simulations.

### Robustness of the Solution

In this work we present a series solution to approximate the solution of the Bethe equation. We have tested the error in this approximation by comparison to a numerical solution, tabulated data, and SRIM calculations. We find excellent agreement over a defined proton energy range applicable to radiotherapy and radionuclide production. One immediate benefit of this solution is that estimate water-equivalent-thickness for radiotherapy applications can be improved, over the empirical Bragg-Kleemann law^19^.

Care must be given in interpretation of our solution. In essence, we advance a simpler solution to the Bethe equation. Although the Bethe equation is routinely used, there are limitations in its use. In practice, there are a number of physical mechanisms which cause deviations from the range predicted by Eq. 6. As is evident in our results, the first type of deviations occurs at low incident energy, where the first-Born approximation reaches its limit. Here we find corrections to the form of the Bethe equation by Barkas or Bloch^6^. In this energy regime the Barkas, Bloch and Shell correction need to be applied. In addition, at high projectile energies, relativistic and density effects and radiative losses need to be taken into account^6^.

In the second category, we find that the distance travelled by the particle along its initial direction is slightly reduced by small deflections caused by scattering. The impact of this is minimal for low *Z* materials, with the ‘detour factor’ of the projected range greater than 99.87% for protons of energy >100 MeV in water^6^ or for metals where the stopping distance is small. Outside of this range, the detour factor becomes substantial, especially for gases^16^ where Schlyer *et al*.^20^ shows that the standard deviation of the probability distribution in scattering angle is related in a complex manner to the local energy of the beam.

In the third category, we find a number of works highlighting the effect of a two-way coupling between the beam and the absorbing material on the irradiation profile. In perhaps the most widely cited series of work, Heselius and his co-workers^21,22^ demonstrate experimentally symmetry-breaking in the beam profile when density of the gas target is stratified due to localized heating. Indeed, this problem is relatively unexplored on the theoretical front with most authors de-coupling the temperature effects^23–26^. In perhaps the most comprehensive study, O’Brien and her co-workers^25,27,28^ couples Monte-Carlo simulation of the beam profile with Reynolds-Averaged Navier-Stokes (RANS)^29^ modelling of the transport equations to estimate the temperature, density and beam profile. Interestingly, with these latter two categories, these deviations are not evident in our result in this range, even with gas.

## Conclusion

We present a simple and easy to implement approximate solution of the Bethe equation with good agreement in the proton energy range of 10 MeV to approximately 200 MeV, applicable for the energy range of proton radiotherapy and medical isotope production. Although tabulated values are available for uniform materials, this solution methodology dramatically reduces the effort to estimate the range in or the dose distribution for mixed media, i.e. composite bodies or multi-layer materials, or estimating water equivalent thickness. Within solid target design, it is now possible to combine computational fluid dynamics codes with our method to estimate internal heating; instead of coupling these with Monte-Carlo packages. Our approach can also easily be coupled with fluid materials for reduced-order modeling of the interaction caused by irradiation and consequent fluid-dynamic effects. This in turn simplifies and accelerates the solution finding and is therefore a potentially convenient tool to advance radiotherapy and medical isotope-production targetry.
